# Physiological response to lipid peroxidation in ischemia and reperfusion during carotid endarterectomy

**DOI:** 10.1186/1476-511X-9-41

**Published:** 2010-04-21

**Authors:** Sebastiano Banni, Roberto Montisci, Roberto Sanfilippo, Gabriele Finco, Daniela Sanna, Elena Giordano, Elisabetta Murru, Lina Cordeddu, Gianfranca Carta, Donata Banni, Antonio Marchi

**Affiliations:** 1 Università degli Studi di Cagliari - Dipartimento di Biologia Sperimentale - Cittadella, Universitaria - 09042 Monserrato, Cagliari, Italy; 2 Università degli Studi di Cagliari - Servizio di Anestesia e Rianimazione - Policlinico Universitario - 09042 Monserrato, Cagliari, Italy; 3 Università degli Studi di Cagliari - Chirurgia Vascolare e Toracica - Policlinico Universitario - 09042 Monserrato, Cagliari, Italy

## Abstract

**Background:**

In this study we aimed to assess lipid peroxidation during carotid endarterectomy by the formation of PUFA hydroperoxides (PUFAHP) and isoprostanes (IP) and concomitant peroxisomal beta-oxidation as a physiological mechanism to limit their concentration. Two markers of peroxisomal beta oxidation have been evaluated, formation of 2,3 dinor from IP and conjugated esadecadienoic acid (CD 16:2) from peroxisomal beta-oxidation of conjugated linoleic acid (CLA), an unusual fatty acid present in small concentration in our diet and preferentially beta-oxidised in peroxisomes.

The study was conducted on 30 patients undergoing carotid endarterectomy. Blood samplings were performed before, during endarterectomy in the "ischemic phase", and 30 seconds, 30 minutes and 2 hours after reperfusion.

**Results:**

The results showed that PUFAHP increased significantly after 30 min of reperfusion in patients with controlateral stenosis > 50%, and steeply decreased after 2 hour of reperfusion. Interestingly, IP increased in a similar fashion of PUFAHP but never significantly. Both ratios CD16:2/CLA and DIN/IP also increased significantly after 30 min of reperfusion to decrease thereafter.

**Conclusions:**

Our data show that lipid peroxidation takes place only in patients with high controlateral stenosis and within 2 hours occurs a physiological response aimed to decrease IP and PUFAHP by increasing their catabolism in peroxisomes.

## Introduction

Carotid endarterectomy is clinically performed to reduce the incidence of strokes in patients with carotid stenosis >70% [1]. However, surgery has some important complications, although rare, such as major or minor stroke and cerebral hyperperfusion syndrome [1]. This surgical procedure involves ischemia-reperfusion, which may induce neurooxidative stress, by free radicals formation [2-5]. Reperfused brain is particularly sensitive to oxidative stress because of a relatively modest antioxidant defenses, high mitochondrial density and neuronal membrane lipids rich in polyunsaturated fatty acid (PUFA) side chains which are particularly susceptible to free radical attack triggering lipid peroxidation [3]. The primary products of lipid peroxidation are fatty acid hydro(pero)xides among which those derived from linoleic acid are one of the most reliable marker [6]. Fatty acid hydroperoxides, are quite unstable and capable to propagate free radical reactions and thereby extending the damage.

One of the most widely used indirect markers of lipid peroxidation is the detection of 8-iso-PGF2α isoprostane (IP) [7]. Its measurement has been widely employed to a great degree over the last decade to detect and quantify this free radical-mediated process in a variety of biological matrices [7].

The IP is regularly reported to be a non-enzymatic (free radical-induced) isoprostanes, although cyclooxygenase-2-dependent formation of small amounts of IP has been reported [8].

IP may be further metabolized by a combination of β-oxidation, double bond reduction, alcohol group oxidation, ω-hydroxylation and ω-oxidation, leading to a myriad of metabolites, which are eventually excreted in the urine [9-11].

One of the mechanism of protection against fatty acid hydroperoxides and IP is their catabolism in peroxisomes [12,13]. Peroxisomal beta oxidation occurs when fatty acids are not a good substrate for mitochondria [14]. These unusual fatty acids include eicosanoids, isoprostanes, fatty acid hydroperoxides. Recently, we showed that another unusual PUFA, conjugated linoleic acid (CLA), with peculiar biological activities [15], widely present in our diet because relatively abundant in dairy products, is preferentially beta oxidized in peroxisomes in experimental animals and humans [16]. Furthermore, we demonstrated that in neurons and astrocytes, both in vivo and in vitro, CLA is well absorbed and avidly beta oxidesed in peroxisomes yielding a conjugated metabolite, hexadecadienoic acid (CD 16:2) [17]. In addition, in different experimental models we have some evidence that during oxidative stress, the peroxisomal beta oxidation of CLA increased [18]. With this study we aimed to verify the degree and at what extent lipid peroxidation occurs in ischemia and reperfusion during carotid endarterectomy, and if a physiological response occurs in terms of increased peroxisomal beta oxidation aimed to degrade potential dangerous lipid peroxidation products. Carotid endarterectomies are performed under regional anaesthesia because of easy and reliable monitoring of cerebral perfusion.

## Materials and methods

30 patients were admitted to the Hospital of the University of Cagliari with a carotid stenosis >70%: 17 patients (8 women and 9 men, age 74,8 ± 4,1) presented with a controlateral carotid stenosis >50%; 13 patients (7 women and 6 men, age 71,5 ± 1,5) presented with a controlateral stenosis <50%. Intra-arterial blood pressure, five-lead electrocardiography (leads II and V5) and pulse oximetry were commenced before placement of the anaesthetic block and were continued during the procedure. The patients were all submitted to the block of deep and superficial cervical plexus, in the supine position with the head facing away from the side to be blocked. Regional anaesthesia was achieved using lidocaine solution 2% (10 mL) and ropivacaine 0,75% (15 mL).

**Figure 1:**
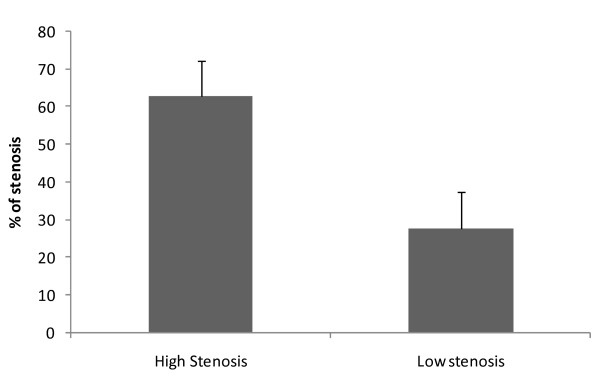
**Percentage of controlateral stenosis of the two groups of patients**.

The 30 surgical patients were divided into two distinct groups according to the degree of stenosis of the internal carotid artery controlateral to the one to be operated. The controlateral carotid was then distinguished into two groups according to the degree of stenosis: up to 40% and greater than 50% (figure 1).

The degree of stenosis was evaluated by Duplex scan and angio CT scan in all cases.

Blood samplings were performed before carotid clamping (p1), during endarterectomy in the "ischemic phase" (p2), and 30 seconds (p3) 30 minutes (p4) and 2 hours (p5) after reperfusion.

All Patients signed informed consensus form during the routine anaesthesiology visit.

Blood samples (3 ml) were taken from the antecubital veins in EDTA containing vials. The blood was immediately centrifuged at 2,000 g for 10 min. Total lipids were then extracted and quantified from the resulting plasma and stored as previously reported [17].

Separation of CLA and its metabolites was carried out with an Agilent 1100 HPLC system (Agilent, Palo Alto, CA) equipped with a diode array detector. A C-18 Inertsil 5 ODS-2 Chrompack column, (Chrompack International BV, Middleburg, the Netherlands), 5 μm particle size, 150 × 4.6 mm, was used with a mobile phase of CH3CN/H2O/CH3COOH (70/30/0.12, v/v/v) at a flow rate of 1.5 ml/min [19]. Conjugated diene unsaturated fatty acids, including PUFA hydroperoxides, were detected at 234 nm. Spectra (195-315 nm) of the eluate were obtained every 1.28 s and were electronically stored. Second-derivative UV spectra of the conjugated diene fatty acids were generated using Phoenix 3D HP Chemstation software (Agilent, Palo Alto, CA). These spectra were taken to confirm the identification of the HPLC peaks [20].

IP and DIN were quantified using HPLC-MS as described in [18].

One way ANOVA with the Tuckey test for post-hoc analyses was applied to evaluate statistical differences between groups.

## Results

**Figure 2:**
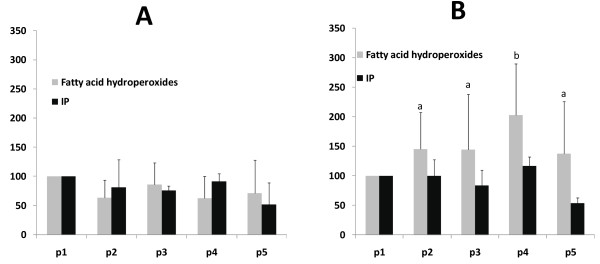
**Changes as percentage of the values of polyunsaturated fatty acids hydroperoxides and Isoprostanes (IP) in plasma of patients with low controlateral stenosis (A) and high controlateral stenosis (B)**. Blood samplings were performed before (p1), during endarterectomy in the "ischemic phase" (p2), and 30 seconds (p3) 30 minutes (p4) and 2 hours (p5). Different letters denote significant differences (p < 0.05).

As it can be seen from figure 2, in patients with high controlateral stenosis, plasma PUFAHP and IP increased after 30' of reperfusion while decreased thereafter, but only PUFAHP significantly.

**Figure 3:**
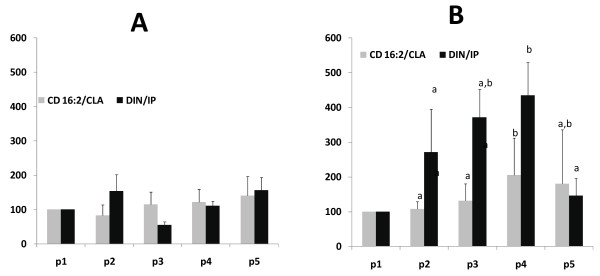
**Changes as percentage of the values of ratios between conjugated hexadecadienoic acid (CD 16:2)and its precursor conjugated linoleic acid (CLA) and 2,3 dinor (DIN) and its precursor isoprostanes (IP) as a measure of peroxisomal beta oxidation, in plasma of patients with low controlateral stenosis (A) and high controlateral stenosis (B)**. Blood samplings were performed before (p1), during endarterectomy in the "ischemic phase" (p2), and 30 seconds (p3) 30 minutes (p4) and 2 hours (p5).

The ratios CD 16:2/CLA and DIN/IP (figure 3) increased significantly in a similar fashion as PUFAHP and IP. Peculiarly, the ratio DIN/IP increased more steeply which seems to prevent a significant increase of IP. In patients with low controlateral stenosis no changes of all parameters measured were detected. All the other plasma fatty acid measured didn't change significantly (data not shown).

## Discussion

A condition of oxidative stress or other specific stimuli can result in two different scenarios. Free radicals generated during oxidative stress may attack the esterified PUFAs, eventually resulting in the formation of a range of esterified PUFA hydroperoxides. The PUFA hydroperoxides may then be released from the phospholipid through the action of phospholipases. The released PUFAHP can then be a substrate for peroxisomal beta-oxidation and activate nuclear receptors such as peroxisome proliferators activated receptor (PPAR) alpha, a transcription factor for a series of enzymes involved in fatty acid metabolism including peroxisomal beta oxidation [14]. The overall results is an increased peroxisomal beta-oxidation which has a physiological meaning in terms of decreasing the levels of lipid peroxidation products potentially dangerous for cells.

A similar mechanism has been shown to occur for IP, which is beta oxidised in peroxisomes to DIN [18].

CLA is a naturally occurring dietary fatty acid good substrate for peroxisomal beta-oxidation yielding an intermediate product CD 16:2 [19]. We have previously shown that peroxisomal beta oxidation is particularly efficient in brain [17]. Furthermore, we have shown that CD 16:2 is promptly released from cells [19]. It is therefore likely that once formed in the brain CD 16:2 is then released in the blood stream. Thereby CD16:2/CLA as well DIN/IP are good markers of peroxisomal beta-oxidation occurring in peripheral tissues [18].

During ischemia, collateral flow is a cornerstone of cerebral blood flow (CBF) compensation. Pathways of collateral flow include the circle of Willis, extracranial anastomotic channels, and leptomeningeal communication that bridge "watershed" areas between major arteries. During carotid endarterectomy, the risk of ischemia is related to the dependency of the cerebral circulation on the ipsilateral internal carotid artery and the cerebrovascular reserve from the controlateral circulation.

In our conditions of ischemia-reperfusion during endarterectomy, brain is the most likely source of these products. Furthermore, from our data it is clear that the significant increase occurs only 30 min after reperfusion and only in those patients with a controlateral stenosis greater than 50%. Interestingly the concomitant increase of CD16:2/CLA and DIN/IP ratios are suggestive of an increased peroxisomal beta oxidation as a physiological response to oxidative stress resulting in a decrease of PUFAHP and IP 2 hours after reperfusion. It is noteworthy that IP never increased significantly and to this is associated a steeply increase of its catabolism in peroxisomes as demonstrated by the ratio DIN/IP, suggesting an efficient physiological response to IP formation.

Therefore oxidative stress during endarterectomy, occurs in a relatively short frame of time due to the prompt catabolism of lipid peroxidation products. Whether this transient oxidative stress may impair brain function is not clear. However, a nutritional and/or a pharmacological approach to decrease or eliminate this transient oxidative stress in patients with a controlateral stenosis greater than 50% may be beneficial and prevent further brain damage. There might be different ways to increase antioxidant status in those patients, either with a preventive dietary intervention with antioxidants which is preferred whenever is possible, or a direct IV intervention during the surgical procedure.

In this study we hypothesize for the first time that there is another physiological defense system against lipid peroxidation, suggesting that not only antioxidant mechanism may play a role, but also peroxisomal beta oxidation could synergistically help to limit oxidative stress. Probably while antioxidant systems act more on the formation and chain propagation of free radical, peroxisomal beta oxidation is more efficient in decreasing concentration and exposure time to fatty acid hydroperoxides and eicosanoids in tissue cells. Interestingly, in brain where the antioxidant defense is weaker, peroxisomal beta oxidation is more efficient. Future studies on this direction may explain some apparent discrepancies on the lack of efficacy of antioxidant therapy in some disease where oxidative stress is involved [21,22]. Another interesting observation is that increased dietary omega-3 PUFAs failed to enhance oxidative stress, despite the increased membrane PUFAs particularly susceptible to lipid peroxidation [23]. PUFA omega-3 are good ligand of PPARalpha which once activated may increase peroxisomal beta oxidation. Therefore, PUFA omega-3, despite their oxidative susceptibility, may enhance the defense system against lipid peroxidation by activating PPARalpha. This mechanism may be particularly active in the brain where omega-3 are the most represented PUFA family.

Therefore, our data suggest that not only antioxidants may have a beneficial effects but also all those strategies in increasing PUFAHP catabolism may also be efficient, probably working synergistically. We aim to verify, by using our experimental approach, which will be the best strategies to avoid the occurrence of lipid peroxidation in ischemia and reperfusion during endarterectomy under regional anaesthesia.

## Abbreviations

PUFAHP: Polyunsaturated fatty acids; IP: isoprostanes; DIN: 2,3 dinor; CLA: conjugated linoleic acid; CD 16:2: conjugated hexadecadienoic acid.

## Declaration of interests

The authors declare that they have no competing interests.

## Authors' contributions

SB, AM conceived of the study, participated in its design and supervision and drafted the manuscript with the contribution of all Authors; GF, DB, DS, AM carried out Patients evaluation, the anesthesia procedure, blood sampling and clinical analyses; RB, RS, evaluated the inclusion criteria by the degree of stenosis and performed the endarterectomy procedure; EG, EM, LC, GC performed all analyses of oxidative stress and peroxisomal beta oxidation markers, collected all data and made statistical analyses. All authors read and approved the final manuscript.
